# Traumatic Birth Perception and Postpartum Depression Among Postpartum Women in Türkiye: The Indirect Association of Perceived Social Support

**DOI:** 10.3390/healthcare14152390

**Published:** 2026-08-04

**Authors:** Serap Öner

**Affiliations:** Department of Midwifery, Faculty of Health Sciences, Fırat University, Elazığ 23119, Türkiye; soner@firat.edu.tr

**Keywords:** traumatic birth perception, postpartum depression, perceived social support, maternal mental health, indirect association

## Abstract

**Background/Objectives**: Traumatic birth perception has been associated with adverse psychological outcomes during the postpartum period, including postpartum depressive symptoms. Perceived social support may be related to both traumatic birth perception and maternal mental health. However, the associations among these variables remain insufficiently understood. This study aimed to examine the relationships among traumatic birth perception, perceived social support, and postpartum depression in postpartum women in Türkiye and to explore the indirect association of perceived social support in this relationship. **Methods**: This cross-sectional study was conducted with 297 postpartum women in Türkiye using an online survey. Data were collected using a Personal Information Form, the Traumatic Birth Perception Scale, the Perceived Social Support Scale (F-SozU K-3), and the Edinburgh Postnatal Depression Scale. Data were analyzed using descriptive statistics, Pearson correlation analysis, and regression-based indirect association analysis using Hayes’ PROCESS macro. **Results**: Traumatic birth perception was positively associated with postpartum depression (*p* < 0.001), whereas perceived social support was negatively associated with postpartum depression (*p* < 0.001). Traumatic birth perception was also negatively associated with perceived social support (*p* < 0.001). The indirect association analysis indicated that perceived social support was significantly associated with the relationship between traumatic birth perception and postpartum depression. Women reporting higher traumatic birth perception tended to report lower perceived social support and higher postpartum depressive symptoms. **Conclusions**: The findings suggest that traumatic birth perception, perceived social support, and postpartum depression are significantly interrelated among postpartum women. Higher levels of perceived social support were associated with lower levels of postpartum depressive symptoms. Given the cross-sectional nature of the study, causal inferences cannot be made. Longitudinal studies are needed to clarify the temporal relationships among these variables.

## 1. Introduction

Childbirth is a major biopsychosocial transition that can profoundly influence a woman’s physical, emotional, and social well-being. Although childbirth is commonly perceived as a positive and life-enhancing event, a considerable proportion of women experience childbirth as distressing or traumatic [1,2]. Traumatic birth perception refers to a woman’s subjective appraisal of childbirth as an event involving intense fear, helplessness, loss of control, or perceived threat to her own life or that of her infant [3]. Such perceptions may persist beyond the birth experience itself and have been associated with a range of adverse psychological outcomes, including post-traumatic stress symptoms, impaired maternal well-being, difficulties in mother–infant bonding, and postpartum depressive symptoms [2,3]. Given the potential short- and long-term consequences of traumatic birth experiences, understanding factors associated with traumatic birth perception is important for promoting maternal mental health during the postpartum period.

Traumatic birth perception is not determined solely by events occurring during labor and birth but is also influenced by women’s pre-existing beliefs, expectations, previous experiences, and sociocultural perspectives regarding childbirth. According to cognitive appraisal theories, individuals interpret potentially stressful events through the lens of their prior knowledge, expectations, and perceived ability to cope with the situation. Consequently, women who perceive childbirth as unpredictable, dangerous, painful, or beyond their control may be more likely to interpret the experience as traumatic [1,3,4]. Previous studies have demonstrated that traumatic birth perception is associated with a range of factors, including sociodemographic characteristics, obstetric experiences, personality traits, fear of childbirth, and childbirth-related knowledge [2]. These perceptions may subsequently influence women’s emotional adjustment during the postpartum period as well as their attitudes toward future pregnancies and childbirth experiences.

Traumatic birth experiences have been consistently associated with a range of adverse psychological outcomes during the postpartum period, among which postpartum depression is one of the most common and clinically significant. Postpartum depression is a mood disorder that may occur within the first year after childbirth and has important consequences not only for maternal mental health but also for mother–infant interaction, infant development, family relationships, and overall family functioning [5,6]. Globally, postpartum depression is estimated to affect approximately 10–20% of women during the postpartum period [4,5]. More broadly, postpartum recovery is a multidimensional process encompassing physical, psychological, and social well-being, and patient-reported outcome measures have increasingly been used to evaluate recovery following childbirth [7,8]. The development of postpartum depression is multifactorial, involving a complex interaction of biological, psychological, obstetric, and social determinants. Therefore, identifying modifiable risk factors remains a priority for maternal mental health research and clinical practice. Previous research indicates that traumatic or distressing childbirth experiences are associated with increased postpartum depressive symptoms [3,6]. Social support may buffer the psychological effects of stressful experiences and reduce vulnerability to adverse mental health outcomes [9]. Negative cognitive appraisals of childbirth, feelings of fear and loss of control, and distressing birth memories may contribute to poorer psychological adjustment after birth, thereby increasing vulnerability to postpartum depressive symptoms [1].

The postpartum period is a complex and multidimensional stage characterized not only by physical recovery following childbirth but also by substantial psychological, emotional, and social adjustments. During this period, women may experience a variety of challenges, including physical discomfort, sleep disturbances, changing family roles, and increased caregiving responsibilities, all of which may influence their overall well-being [7,8]. Consequently, social relationships and perceived support constitute important psychosocial resources during the transition to motherhood [10,11].

Perceived social support refers to an individual’s belief that emotional, informational, and practical assistance is available from family members, friends, partners, and other significant individuals when needed [9,10]. In the context of childbirth, inadequate social support has also been associated with postpartum post-traumatic stress symptoms [12]. A large body of evidence suggests that perceived social support is associated with better psychological adjustment, reduced stress, and improved mental health outcomes across different life stages. During the postpartum period, women who perceive higher levels of social support generally report better emotional well-being and lower levels of psychological distress than those with inadequate support [11,13,14]. Therefore, perceived social support is increasingly recognized as an important factor associated with maternal mental health during the postpartum period.

Perceived social support has been consistently associated with better psychological outcomes following stressful and potentially traumatic life events. According to the stress-buffering hypothesis, social support may reduce the adverse psychological consequences of stressful experiences by enhancing coping resources and promoting emotional resilience [9]. In the context of childbirth, social support may be particularly important because women often rely on partners, family members, and healthcare professionals for emotional reassurance, practical assistance, and guidance during the transition to motherhood. Within the Turkish context, traumatic birth perception has been operationalized using the Traumatic Birth Perception Scale, a culturally developed instrument designed to assess women’s subjective perception of childbirth as traumatic [15]. The brief F-SozU K-3 provides an economical measure of perceived social support and has been validated in its original form and subsequently adapted for use in a Turkish sample [16,17].

Previous studies have demonstrated that lower levels of perceived social support are associated with higher levels of postpartum depressive symptoms, increased psychological distress, and poorer maternal well-being [11,13,14]. Similarly, inadequate social support has been associated with higher levels of childbirth-related traumatic stress and post-traumatic stress symptoms following childbirth [12,13].

Previous research has demonstrated associations between traumatic childbirth experiences, perceived social support, and postpartum depressive symptoms. Studies have shown that traumatic birth experiences are associated with poorer maternal psychological outcomes, whereas higher levels of perceived social support are generally associated with better emotional adjustment during the postpartum period [2,3,13,14]. However, most existing studies have examined these variables separately or have focused primarily on direct associations between them. Consequently, there remains limited evidence regarding how perceived social support may be associated with the relationship between traumatic birth perception and postpartum depressive symptoms, particularly among postpartum women in Türkiye, where postpartum depression and cesarean birth rates remain important public health concerns.

Understanding these interrelationships may have important implications for maternal mental health care. If perceived social support is associated with both traumatic birth perception and postpartum depressive symptoms, interventions aimed at strengthening social support resources may contribute to improved psychological well-being during the postpartum period. Furthermore, previous research suggests that perceived social support may be associated with postpartum depressive symptoms partly through difficulties in emotion regulation [14]. Building on this literature, the present study explores the associations among traumatic birth perception, perceived social support, and postpartum depression and examines the indirect association of perceived social support in this relationship.

The primary objective of this study was to examine the associations among traumatic birth perception, perceived social support, and postpartum depression among postpartum women in Türkiye. The secondary objective was to explore the indirect association of perceived social support in the relationship between traumatic birth perception and postpartum depressive symptoms.

## 2. Materials and Methods

### 2.1. Study Design

This study employed a cross-sectional design to examine the associations among traumatic birth perception, perceived social support, and postpartum depression and to explore the indirect association of perceived social support in this relationship.

### 2.2. Participants and Sampling

The target population of this study consisted of women who had given birth within the previous 12 months in Türkiye. Participants were recruited using a non-probability snowball sampling approach through online platforms, including Instagram and WhatsApp. The survey link was disseminated through social media networks and participants were encouraged to share the survey with other eligible women.

A total of 324 responses were initially received. During the data screening process, questionnaires with substantial missing data, inconsistent response patterns, duplicate submissions, or incomplete scale responses were excluded. After data cleaning, 297 questionnaires were retained for the final analyses.

Because a non-random sampling strategy was used, the findings should be interpreted with caution and may not be generalizable to all postpartum women in Türkiye.

The minimum required sample size was calculated using G*Power version 3.1 for multiple regression analysis. Assuming a medium effect size (f^2^ = 0.15), a significance level of 0.05, and a statistical power of 0.80, the minimum required sample size was estimated to be 150 participants. The final sample of 297 women exceeded this requirement and was considered sufficient to detect medium-sized associations among the study variables.

### 2.3. Inclusion and Exclusion Criteria

Women who were aged 18 years or older, had given birth within the past year, were able to read and understand Turkish, and provided informed consent were eligible for participation.

### 2.4. Data Collection Instruments

#### 2.4.1. Personal Information Form

The Personal Information Form was developed by the researchers based on the literature and included 15 items assessing participants’ sociodemographic and obstetric characteristics, such as age, education level, employment status, income level, type of delivery, and number of pregnancies.

#### 2.4.2. Traumatic Birth Perception Scale

The Traumatic Birth Perception Scale (TBPS) was developed by Yalnız et al. [15] to assess women’s perceptions of childbirth as a traumatic experience. The scale consists of 13 items rated on an 11-point scale ranging from 0 to 10, with higher scores indicating higher levels of traumatic birth perception. During the original scale development study, the instrument demonstrated satisfactory psychometric properties, including a Kaiser–Meyer–Olkin value of 0.857, a significant Bartlett’s test of sphericity, and a Cronbach’s α coefficient of 0.895. The total score ranges from 0 to 130, with higher scores reflecting higher levels of traumatic birth perception. In the present study, the Cronbach’s α coefficient was 0.948.

#### 2.4.3. Perceived Social Support Scale (F-SozU K-3)

The Perceived Social Support Questionnaire (F-SozU K-3) was originally developed by Petersen et al. [16] as a brief three-item measure of perceived social support. Each item is scored on a 5-point Likert scale, with higher scores indicating higher levels of perceived social support. The Turkish version demonstrated acceptable psychometric properties, including satisfactory construct validity and internal consistency (Cronbach’s α = 0.82). In the present study, Cronbach’s α coefficient was 0.822.

#### 2.4.4. Edinburgh Postnatal Depression Scale (EPDS)

The Edinburgh Postnatal Depression Scale (EPDS) is a 10-item instrument developed by Cox et al. (1987) [18] to assess postpartum depression. Items are scored between 0 and 3, and higher total scores indicate a higher risk of depression. The Turkish validity and reliability study was conducted by Engindeniz et al. (1996) [19], with a cut-off score of 13. In this study, the Cronbach’s α coefficient was 0.82. The Turkish version of the EPDS demonstrated satisfactory validity and reliability, with a recommended cut-off score of 13 for identifying women at risk of postpartum depression.

### 2.5. Data Collection Procedure

Data were collected between 10 March and 22 April 2026 using an online questionnaire. Participants were informed about the purpose of the study, and informed consent was obtained prior to participation. Only individuals who approved the consent form were included. Data were collected anonymously and handled in accordance with confidentiality principles. The survey was administered online and anonymous participation was permitted. Prior to analysis, the dataset was reviewed for completeness and consistency. Questionnaires with incomplete responses, substantial missing data, duplicate submissions, or obvious response inconsistencies were excluded from the final analyses. Formal attention-check items were not included in the survey.

### 2.6. Statistical Analysis

Data were analyzed using SPSS version 25. Descriptive statistics were presented as frequencies, percentages, means, and standard deviations. Normality was assessed using the Kolmogorov–Smirnov test along with skewness and kurtosis values. Given the relatively large sample size, normal distribution assumptions were primarily evaluated based on skewness and kurtosis. Skewness and kurtosis values for all continuous variables were within the acceptable range of ±2, indicating no substantial deviations from normality.

Pearson correlation analysis was used to examine relationships between variables. Multiple linear regression analysis was conducted to examine the association between traumatic birth perception and postpartum depression. Age, education level, employment status, income level, number of pregnancies, type of delivery, pregnancy planning, and pregnancy-related complications were included as covariates based on prior literature addressing postpartum depressive symptoms, acute stress, childbirth-related beliefs, and associated sociodemographic and obstetric factors [20,21,22,23].

Categorical covariates were entered into the regression models using indicator coding. The reference categories were primary school education, employed status, income lower than expenses, vaginal delivery, planned pregnancy, and absence of pregnancy-related complications. Regression assumptions and model quality were evaluated using residual histograms and Q–Q plots, residual skewness and kurtosis, residual-versus-fitted plots, the Breusch–Pagan test for heteroscedasticity, adjusted generalized variance inflation factors for multicollinearity, and studentized residuals and Cook’s distance for potentially influential observations. When heteroscedasticity was detected, HC3 heteroscedasticity-robust standard errors were examined in sensitivity analyses.

The indirect association of perceived social support was explored using Hayes’ PROCESS macro (Model 4). A bootstrap method with 5000 resamples was used to estimate indirect effects and calculate 95% confidence intervals (CIs). Indirect effects were considered statistically significant when the confidence interval did not include zero. Statistical significance was set at *p* < 0.05.

To provide a descriptive assessment of potential selection bias, the mean maternal age and cesarean delivery rate in the sample were compared with the most recent official national statistics. No formal statistical testing or weighting was performed because individual-level population data were not available.

### 2.7. Ethical Considerations

Ethical approval was obtained from the relevant institutional review board (Decision No: 2026/04-19; Date: 5 March 2026). The study was conducted in accordance with the Declaration of Helsinki. Informed consent was obtained from all participants, and confidentiality and data protection principles were strictly maintained.

## 3. Results

The sociodemographic and obstetric characteristics of the participants are presented in Table 1. The study was completed with 297 women. The mean age of the participants was 29.9 ± 6.0 years (range: 22–48). Of the participants, 57.5% had a high school or higher level of education, and 54.2% were not employed. Additionally, 51.5% had a vaginal delivery, and 76.8% reported that their pregnancy was planned.

The descriptive statistics and reliability coefficients of the study variables are presented in Table 2. The mean total scores were 59.1 ± 33.6 for the Traumatic Birth Perception Scale, 8.9 ± 3.8 for the Perceived Social Support Scale, and 10.7 ± 5.5 for the Edinburgh Postnatal Depression Scale. Cronbach’s α coefficients ranged from 0.815 to 0.948, indicating good internal consistency (Table 2).

To provide context regarding potential selection bias, selected sample characteristics were descriptively compared with recent national indicators. The mean age of the participants was similar to the national mean age of women giving birth in Türkiye in 2025 (29.9 vs. 29.4 years). However, the proportion of cesarean deliveries in the sample was lower than the national cesarean delivery rate reported for 2024 (48.5% vs. 61.2%). Thus, although the sample was broadly comparable to the national population in terms of maternal age, women who delivered by cesarean section may have been underrepresented [24,25].

Correlation analysis revealed a moderate positive relationship between traumatic birth perception and postpartum depression (r = 0.49, *p* < 0.001), a strong negative relationship between perceived social support and postpartum depression (r = −0.72, *p* < 0.001), and a moderate negative relationship between traumatic birth perception and perceived social support (r = −0.39, *p* < 0.001) (Table 3).

The complete results of the adjusted regression models are presented in Table 4. In the total-effect model, traumatic birth perception was positively associated with postpartum depressive symptoms after adjustment for the covariates (B = 0.044, SE = 0.008, β = 0.268, *p* < 0.001). The model explained 44.1% of the variance in postpartum depressive symptoms (adjusted R^2^ = 0.415). In the mediator model, traumatic birth perception was negatively associated with perceived social support (B = −0.024, SE = 0.006, β = −0.206, *p* < 0.001), with the model explaining 33.4% of the variance in perceived social support (adjusted R^2^ = 0.304). In the final outcome model, both traumatic birth perception (B = 0.027, SE = 0.007, β = 0.161, *p* < 0.001) and perceived social support (B = −0.743, SE = 0.065, β = −0.517, *p* < 0.001) remained independently associated with postpartum depressive symptoms. This model explained 61.9% of the variance in postpartum depressive symptoms (adjusted R^2^ = 0.600). The bootstrapped indirect association was statistically significant (B = 0.018, BootSE = 0.005, 95% CI: 0.008 to 0.028).

Examination of the regression diagnostics indicated that residual skewness and kurtosis values were within ±1 across all models. The Breusch–Pagan test did not indicate heteroscedasticity in the total-effect or mediator models (*p* = 0.869 and *p* = 0.510, respectively), whereas mild heteroscedasticity was detected in the final outcome model (*p* = 0.037). Therefore, an HC3 robust standard-error sensitivity analysis was performed; the associations of traumatic birth perception and perceived social support with postpartum depressive symptoms remained statistically significant (both *p* < 0.001). The adjusted generalized variance inflation factors ranged from 1.14 to 3.12. No observation had a Cook’s distance greater than 1, indicating the absence of highly influential cases.

The indirect association of traumatic birth perception with postpartum depressive symptoms through perceived social support was statistically significant (B = 0.018, BootSE = 0.005, 95% CI [0.008, 0.028]). Because the bootstrap confidence interval did not include zero, the indirect association was considered statistically significant. These findings suggest that perceived social support may be indirectly associated with the relationship between traumatic birth perception and postpartum depressive symptoms.

## 4. Discussion

This study identified significant associations among traumatic birth perception, perceived social support, and postpartum depressive symptoms. Higher levels of traumatic birth perception were associated with higher levels of postpartum depressive symptoms, whereas higher levels of perceived social support were associated with lower levels of postpartum depressive symptoms. Furthermore, the findings suggested that perceived social support may be indirectly associated with the relationship between traumatic birth perception and postpartum depressive symptoms. These results highlight the potential importance of social support in understanding maternal psychological well-being during the postpartum period.

A significant positive association was found between traumatic birth perception and postpartum depressive symptoms, which is consistent with previous studies reporting associations between traumatic childbirth experiences and poorer maternal mental health outcomes. Traumatic birth perception is commonly characterized by emotional responses such as fear, loss of control, and helplessness during childbirth and has been associated with increased psychological distress during the postpartum period [1,2]. Previous studies have also reported that negative cognitive appraisals of childbirth are associated with higher levels of postpartum depressive symptoms and poorer psychological adjustment following birth [3,6]. Consistent with these findings, the results of the present study indicate that women reporting higher levels of traumatic birth perception also reported higher levels of postpartum depressive symptoms.

The strong negative association observed between perceived social support and postpartum depressive symptoms is consistent with previous research examining maternal mental health during the postpartum period. Perceived social support has been widely associated with better psychological well-being, lower levels of stress, and more favorable maternal mental health outcomes [9,10,14]. In the postpartum period, women reporting higher levels of perceived social support generally report lower levels of psychological distress and depressive symptoms than those with lower levels of support [11,13,14]. Previous studies have also reported associations between higher perceived social support and lower levels of childbirth-related traumatic stress and postpartum psychological symptoms [12,13]. The findings of the present study are consistent with this body of literature and further support the observed association between perceived social support and postpartum depressive symptoms.

One of the notable findings of this study was the significant indirect association of perceived social support in the relationship between traumatic birth perception and postpartum depressive symptoms. The observed pattern of associations indicated that women reporting higher levels of traumatic birth perception tended to report lower levels of perceived social support, while lower perceived social support was associated with higher levels of postpartum depressive symptoms. These findings are consistent with theoretical perspectives suggesting that social support is closely linked to psychological well-being following stressful life events [9,10]. Previous studies have similarly reported that perceived social support is associated with lower levels of psychological distress and postpartum depressive symptoms [11,13,14]. Although causal inferences cannot be drawn from the present cross-sectional design, the findings suggest that perceived social support may be an important factor associated with the relationship between traumatic birth perception and postpartum depressive symptoms.

The continued statistical significance of the direct association between traumatic birth perception and postpartum depressive symptoms suggests that the relationship between these variables is multifaceted and may involve factors beyond perceived social support. Childbirth-related experiences are influenced by a wide range of psychological, social, and contextual factors, and it is unlikely that any single variable fully explains postpartum psychological outcomes. Previous studies have similarly reported associations among perceived social support, psychological distress, and postpartum depressive symptoms [13,14]. Social support has also been associated with postpartum recovery quality among women in at-risk age groups [26]. The findings of the present study are consistent with this literature and suggest that perceived social support may represent one of several factors associated with postpartum depressive symptoms.

The findings of this study highlight the potential importance of social support during childbirth and the postpartum period. Healthcare professionals may contribute to more positive childbirth experiences by providing supportive, empathetic, and woman-centered care throughout the perinatal period. In addition, efforts aimed at strengthening family and social support resources may be beneficial for maternal psychological well-being during the postpartum period. Given the observed associations between perceived social support and postpartum depressive symptoms, future interventions and longitudinal studies are warranted to further examine these relationships.

Overall, the findings suggest that perceived social support may represent an important factor associated with the relationship between traumatic birth perception and postpartum depressive symptoms. These results contribute to the growing body of literature examining psychosocial factors related to maternal mental health during the postpartum period.

## 5. Limitations

This study has several limitations. First, due to its cross-sectional design, temporal relationships between variables could not be established and causal inferences cannot be drawn. Second, data were collected through an online survey using a non-probability snowball sampling approach, which may have introduced selection bias and limited the representativeness of the sample. Consequently, the findings may not be generalizable to all postpartum women in Türkiye.

Third, all study variables were assessed using self-report measures administered at a single time point. Therefore, the findings may have been influenced by common method bias, recall bias, and social desirability bias. In addition, formal attention-check items were not included in the online survey, which may have limited the assessment of response validity.

Finally, the study included a limited number of demographic, obstetric, and psychosocial variables. Potentially relevant factors, such as psychiatric history, quality of partner support, stressful life events, and other contextual influences on postpartum mental health, were not assessed. Future longitudinal studies incorporating a broader range of psychosocial variables are needed to further clarify the relationships among traumatic birth perception, perceived social support, and postpartum depressive symptoms.

Although the mean maternal age in the sample was close to the corresponding national figure, the lower proportion of cesarean deliveries and the use of online snowball sampling indicate potential selection bias. Women with limited digital access or lower willingness to participate in online research may have been underrepresented. Therefore, the findings should not be considered fully representative of all postpartum women in Türkiye.

## 6. Conclusions

This study identified significant associations among traumatic birth perception, perceived social support, and postpartum depressive symptoms among postpartum women in Türkiye. Higher levels of traumatic birth perception were associated with higher levels of postpartum depressive symptoms, whereas higher levels of perceived social support were associated with lower levels of depressive symptoms. In addition, the findings suggested that perceived social support may be indirectly associated with the relationship between traumatic birth perception and postpartum depressive symptoms.

These findings highlight the potential importance of social support in understanding maternal psychological well-being during the postpartum period. Healthcare professionals may contribute to more positive childbirth experiences by providing supportive, empathetic, and woman-centered care throughout pregnancy, childbirth, and the postpartum period. In addition, efforts aimed at strengthening family and social support resources may be beneficial for maternal psychological well-being.

Future longitudinal and prospective studies are needed to further examine the relationships among traumatic birth perception, perceived social support, and postpartum depressive symptoms and to better understand the psychosocial factors associated with maternal mental health outcomes.

## Figures and Tables

**Table 1:** Sociodemographic and obstetric characteristics of participants (n = 297).

Variable	$\bar{x}\;$ ± SD	Median (Min–Max)
Age	29.9 ± 6.00	22–48
	**n**	**%**
**Education status**		
Primary school	33	11.1
Secondary school	93	31.3
High school	61	20.5
University	39	13.1
Postgraduate	71	23.9
**Employment status**		
Employed	136	45.8
Not employed	161	54.2
**Types of birth**		
Vaginal birth	153	51.5
Cesarean section	144	48.5
**Pregnancy planning**		
Planned	228	76.8
Unplanned	69	23.2

**Table 2:** Mean scores and reliability coefficients of study variables.

Variable	Mean ± SD	Min–Max	Cronbach’s α
Traumatic Birth Perception	59.1 ± 33.6	4–124	0.948
Perceived Social Support	8.9 ± 3.8	3–15	0.822
Postpartum Depression (EPDS)	10.7 ± 5.5	0–24	0.815

**Table 3:** Correlations between study variables.

Variable	1	2	3
1. Traumatic Birth Perception	1		
2. Perceived Social Support	−0.393 **	1	
3. Postpartum Depression	0.490 **	−0.717 **	1

Note **: *p* < 0.01.

**Table 4:** Complete adjusted regression models examining the indirect association of perceived social support. Panel (**A**). Total-effect model: Outcome—postpartum depressive symptoms. Panel (**B**). Mediator model: Outcome—perceived social support. Panel (**C**). Final outcome model: Outcome—postpartum depressive symptoms.

(**A**)						
**Predictor**	**B**	**SE**	**β**	**t**	***p***	**95% CI**
Constant	11.806	2.950	—	4.002	<0.001	[5.999, 17.613]
Traumatic birth perception	0.044	0.008	0.268	5.263	<0.001	[0.028, 0.061]
Age	0.077	0.076	0.085	1.019	0.309	[−0.072, 0.227]
Education: secondary school	−5.002	1.795	−0.419	−2.786	0.006	[−8.536, −1.469]
Education: high school	−7.594	1.978	−0.555	−3.839	<0.001	[−11.489, −3.700]
Education: university	−5.425	2.962	−0.331	−1.831	0.068	[−11.256, 0.406]
Education: postgraduate	−5.156	2.162	−0.397	−2.385	0.018	[−9.412, −0.901]
Not employed	0.631	1.539	0.057	0.410	0.682	[−2.398, 3.661]
Income equal to expenses	4.927	1.048	0.370	4.702	<0.001	[2.864, 6.989]
Income greater than expenses	5.636	3.313	0.083	1.701	0.090	[−0.884, 12.157]
Number of pregnancies	−3.172	0.670	−0.421	−4.733	<0.001	[−4.491, −1.853]
Cesarean delivery	0.212	1.483	0.019	0.143	0.886	[−2.707, 3.131]
Unplanned pregnancy	−0.194	1.474	−0.015	−0.132	0.895	[−3.096, 2.707]
Pregnancy-related complication	3.203	1.516	0.243	2.113	0.036	[0.219, 6.187]
(**B**)						
**Predictor**	**B**	**SE**	**β**	**t**	***p***	**95% CI**
Constant	6.802	2.240	—	3.036	0.003	[2.392, 11.211]
Traumatic birth perception	−0.024	0.006	−0.206	−3.712	<0.001	[−0.036, −0.011]
Age	−0.005	0.058	−0.008	−0.092	0.927	[−0.119, 0.108]
Education: secondary school	2.494	1.363	0.300	1.830	0.068	[−0.189, 5.178]
Education: high school	5.034	1.502	0.528	3.351	<0.001	[2.077, 7.991]
Education: university	4.560	2.249	0.400	2.027	0.044	[0.132, 8.987]
Education: postgraduate	3.163	1.642	0.350	1.927	0.055	[−0.068, 6.395]
Not employed	0.193	1.169	0.025	0.165	0.869	[−2.108, 2.493]
Income equal to expenses	−2.239	0.796	−0.242	−2.815	0.005	[−3.805, −0.673]
Income greater than expenses	−4.413	2.515	−0.094	−1.754	0.080	[−9.364, 0.539]
Number of pregnancies	1.616	0.509	0.308	3.176	0.002	[0.615, 2.618]
Cesarean delivery	−0.373	1.126	−0.048	−0.331	0.741	[−2.589, 1.844]
Unplanned pregnancy	0.121	1.119	0.013	0.108	0.914	[−2.083, 2.324]
Pregnancy-related complication	−2.867	1.151	−0.313	−2.491	0.013	[−5.133, −0.601]
(**C**)						
**Predictor**	**B**	**SE**	**β**	**t**	***p***	**95% CI**
Constant	16.857	2.480	—	6.797	<0.001	[11.975, 21.739]
Traumatic birth perception	0.027	0.007	0.161	3.742	<0.001	[0.013, 0.041]
Perceived social support	−0.743	0.065	−0.517	−11.467	<0.001	[−0.870, −0.615]
Age	0.073	0.063	0.081	1.169	0.243	[−0.050, 0.197]
Education: secondary school	−3.150	1.494	−0.264	−2.108	0.036	[−6.091, −0.209]
Education: high school	−3.856	1.669	−0.282	−2.310	0.022	[−7.141, −0.571]
Education: university	−2.039	2.469	−0.124	−0.826	0.410	[−6.898, 2.820]
Education: postgraduate	−2.807	1.800	−0.216	−1.559	0.120	[−6.351, 0.737]
Not employed	0.774	1.273	0.070	0.608	0.544	[−1.732, 3.281]
Income equal to expenses	3.264	0.879	0.245	3.714	<0.001	[1.534, 4.994]
Income greater than expenses	2.359	2.755	0.035	0.856	0.393	[−3.065, 7.783]
Number of pregnancies	−1.971	0.564	−0.262	−3.495	<0.001	[−3.082, −0.861]
Cesarean delivery	−0.065	1.227	−0.006	−0.053	0.958	[−2.480, 2.350]
Unplanned pregnancy	−0.105	1.220	−0.008	−0.086	0.932	[−2.505, 2.296]
Pregnancy-related complication	1.074	1.268	0.082	0.847	0.398	[−1.422, 3.569]

Panel (A) Model summary: R^2^ = 0.441; adjusted R^2^ = 0.415; F(13, 283) = 17.167; *p* < 0.001; RMSE = 4.238. Panel (B) Model summary: R^2^ = 0.334; adjusted R^2^ = 0.304; F(13, 283) = 10.926; *p* < 0.001; RMSE = 3.218. Panel (C) Model summary: R^2^ = 0.619; adjusted R^2^ = 0.600; F(14, 282) = 32.683; *p* < 0.001; RMSE = 3.506. Note: n = 297. B, unstandardized regression coefficient; SE, standard error; β, standardized regression coefficient; CI, confidence interval; RMSE, root mean square error. Reference categories were primary school education, employed status, income lower than expenses, vaginal delivery, planned pregnancy, and absence of pregnancy-related complications. Conventional ordinary least-squares standard errors are presented. An HC3 robust standard-error sensitivity analysis for the final outcome model did not alter the statistical significance of the focal predictors. The bootstrapped indirect association was B = 0.018, BootSE = 0.005, 95% CI [0.008, 0.028] based on 5000 bootstrap samples.

## Data Availability

The data presented in this study are available on request from the corresponding author. The data are not publicly available due to ethical considerations and protection of participant confidentiality.
